# Effect of glazing application side and mechanical cycling on the biaxial flexural strength and Weibull characteristics of a Y-TZP ceramic

**DOI:** 10.1590/1678-7757-2020-0438

**Published:** 2020-09-28

**Authors:** Carolina Machado Martinelli LOBO, Sâmia Carolina Mota Cavalcanti SACORAGUE, Nathalia Ramos da SILVA, Anna Karina Figueiredo COSTA, Larissa Marcia Martins ALVES, Marco Antônio BOTTINO, Mutlu ÖZCAN, Rodrigo Othávio de Assunção e SOUZA, Renata Marques de MELO

**Affiliations:** 1 Universidade Estadual Paulista Júlio de Mesquita Filho Instituto de Ciência e Tecnologia Departamento de Materiais Dentários e Prótese São José dos CamposSão Paulo Brasil Universidade Estadual Paulista Júlio de Mesquita Filho (UNESP), Instituto de Ciência e Tecnologia, Departamento de Materiais Dentários e Prótese, São José dos Campos, São Paulo, Brasil.; 2 Universidade Federal do Rio Grande do Norte Departamento de Odontologia NatalRio Grande do Norte Brasil Universidade Federal do Rio Grande do Norte (UFRN), Departamento de Odontologia, Natal, Rio Grande do Norte, Brasil.; 3 University of Zurich Center for Dental and Oral Medicine Division of Dental Biomaterials Zurich Switzerland University of Zurich, Center for Dental and Oral Medicine, Division of Dental Biomaterials, Clinic for Reconstructive Dentistry, Zurich, Switzerland.

**Keywords:** Ceramics, Dental stress analysis, Flexural Strength, Y-TZP

## Abstract

Glaze application on monolithic zirconia (Y-TZP) can be a practical approach to improve the mechanical properties of this material.

**Objective:**

Our study evaluated the effect of glazing side and mechanical cycling on the biaxial flexure strength (BFS) of a Y-TZP.

**Methodology:**

Eighty sintered Y-TZP discs (Ø:12 mm; thickness: 1.2 mm - ISO 6872) were produced and randomly assigned into eight groups (n=10), according to the factors “glazing side” (control – no glazing; GT – glaze on tensile side; GC – glaze on compression side; GTC – glaze on both sides) and “mechanical aging” (non-aged and aged, A – mechanical cycling: 1.2×106, 84 N, 3 Hz, under water at 37°C). Specimens were subjected to BFS test (1 mm/min; 1,000 Kgf load cell) and fractured surfaces were analyzed by stereomicroscopy and SEM. Hsueh’s rigorous solutions were used to estimate the stress at failure of glazed specimens. Two-way ANOVA, Tukey’s test (5%), and Weibull analysis were performed.

**Results:**

The “glazing side”, “mechanical aging” and the interaction of the factors were significant (p<0.05). Groups GC (1157.9±146.9 MPa), GT (1156.1±195.3 MPa), GTC (986.0±187.4 MPa) and GTC-A (1131.9±128.9 MPa) presented higher BFS than control groups (Tukey, 5%). Hsueh’s rigorous solutions showed that the maximum tensile stress was presented in the bottom of zirconia layer, at the zirconia/glaze interface. Weibull characteristic strength (σ_o_) of the GC was higher than all groups (p<0.05), except to GT, GTC-A and GTC, which were similar among them. The fractography showed initiation of failures from zirconia the tensile side regardless of the side of glaze application and fatigue.

**Conclusion:**

Glazing zirconia applied on both tensile and compression sides improves the flexural strength of Y-TZP, regardless the mechanical aging.

## Introduction

Recent studies have shown that zirconium oxide ceramics present higher mechanical properties,^1,2^ biocompatibility and low bacterial adhesion characteristics.^3,4^ However, considering that the conventional zirconia, first generation of yttria-stabilized tetragonal zirconia polycrystals (Y-TZP), has very low translucence, it requires a glass ceramic veneering application, which can favor the chipping and fracture of veneering ceramic,^5^ mainly due to the low thermal conductivity of zirconia.^6^ Chipping is one of the most predominant failures in bilayer zirconia restorations.^5,7,8^ Therefore, the use of monolithic (translucent) zirconia restorations have increased, ^9-11^ on which surfaces only polishing^12^ or glazing^13,14^have been recommended. Glazing is preferred because it prevents surface damages that may lead to phase transformation from tetragonal to monoclinic and, eventually, to low temperature degradation.^15^

The amount of glaze is negligible compared to that of glass ceramic on a bilayer zirconia restoration. Thus, the residual stresses at the interfaces is probably also very lower. So far, there is evidence that the application of a glass layer with low elastic modulus to zirconia promotes better stress distribution, because the maximum tensile stress is directed to the high-modulus zirconia,^16,17^ improving the mechanical properties of the material.^18^ Glazing has also been applied in the internal surfaces of zirconia crowns to improve bonding to resins.^19,20^ Furthermore, considering that one of the main areas subjected to tensile stress is the internal surface of the crowns,^21^ glaze application in this area can decrease tensile stress of the zirconia.^16^ A low thickness of glaze layer can also be applied under connectors areas of FDPs, especially in clinical situations where there is lack of space for veneering ceramic.

The stress at glaze/zirconia interface can vary depending on the side (tensile and/or compression) of application. Thus, the effect of glazing side on the mechanical properties of monolithic zirconia remains unclear. In the present study, it was evaluated the influence of glaze side application on the biaxial flexure strength (BFS) of 3Y-TZP ceramic, before and after cycling, under the following protocols: glaze on the tensile side, on the compression side and on the tensile and compression sides. The hypotheses were that: 1) The application of glaze on tensile and/or compression sides increase the flexural strength of Y-TZP; 2) The mechanical aging decreases the flexural strength of zirconia regardless the glazing application side.

## Methodology

### Specimen preparation

Blocks of 3Y-TZP (14×15×40 mm, YZ Vita In-Ceram, Vita Zahnfabrik, Bad Säckingen, Germany) were rounded to a 15 mm diameter cylinder using a core drill and copious lubricant. Eighty discs (∅: 15 mm; thickness: 2.30±0.01 mm) were then sectioned using a low-speed drill (Isomet, Buehler, Lake Bluff, IL, USA). The discs were subsequently polished with #600 to #1500 silicon carbide abrasives sand papers.

The specimens were sintered (Zyrcomat, Vita Zahnfabrik, Bad Säckingen, Germany) at 17^o^C/min for 90 min, 1530°C sintering temperature for 120 min; cooling until 400°C, 3.5 h dwell time and 11 h total time, resulting in a final dimensions: diameter: 12 mm; thickness: 1.20 mm - ISO 6872^22^. The discs were randomly divided into eight groups (n=10), according to the factor “glazing side” (4 levels) and “mechanical aging” (2 levels).

### Glazing application

The zirconia discs were ultrasonically cleaned in isopropyl alcohol 9% for 5 min and dried in an oil-free air stream at room temperature. The low-fusing glaze ceramic (Vita Akzent, Vita Zahnfabrik, Bad Säckingen, Germany) was applied to the zirconia discs, according to the following groups:

- Control: no glazing.
- Glaze on tensile side (GT): a low-fusing glaze ceramic layer was applied to one side of the zirconia discs.
- Glaze on compression side (GC): a low-fusing glaze ceramic layer was applied to one side of the specimen.
- Glaze on tensile and compression sides (GTC): a low-fusing glaze ceramic layer was applied on both sides of the discs.

The low-fusing glaze ceramic (Vita Akzent, Vita Zahnfabrik) was applied by means of a brush and sintered in the Vacumat 40T furnace (Vita Zahnfabrik, Bad Säckingen, Germany) as recommended by the manufacturing. The firing schedule was the following: initial temperature: 500^0^C; time at the initial temperature: 4 min; time for temperature elevation: 5 to 15 min; temperature elevation rate: 80^0^C/min; maximum temperature: 920^0^C; and time at the maximum temperature: 1 min.

### Mechanical aging in water

Half of the specimens were stored in water at 37°C for 24 h and the other half were subjected to 1.2 × 10^6^ mechanical cycles at 3 Hz and to a load of 84 N, under immersion in water at 37°C (A) (ER – 1100 Plus, ERIOS Equipamentos Eireli, São Paulo, SP, Brazil). The specimens of the GT group had their treated side placed opposite to the load during mechanical aging. On the other hand, the specimens of the GC group had their treated side facing to the load during mechanical aging.

### Biaxial flexure strength test

The BFS was determined in a piston-on-three-ball configuration using a cross-head speed of 1 mm/min and 1,000 Kgf load cell at room temperature (25°C). A thin acetate film was placed between the specimen and the metallic piston (Ø=1.6 mm) for uniform distribution of the load and the disc-shaped specimens were positioned with the tensile stress on the three support balls (Ø=3.2 mm) positioned 10 mm apart in a triangular position. The thickness of the specimens was measured before and after the test. The BFS of the control groups was estimated according to ISO 6872.^22^

$$\sigma =-0.2387\cdot \frac{P(X-Y)}{b^{2}}$$

σ is the maximum tensile stress (MPa), *P* is the total load causing fracture (N), *b* is the thickness of the fracture’s origin (mm), and *X* and *Y* are estimated according to:

$$X=(1+v)\ln\left(\frac{r_{2}}{r_{3}}\right)+[\frac{\left(1-v\right)}{2}|\left(\frac{r_{2}}{r_{3}}\right)$$

$$Y=(1+v)\left[1+\ln\left(\frac{r}{r_{3}}\right)\right]+(1-v)\left(\frac{r_{1}}{r_{3}}\right)$$

*v* is Poisson’s ratio (0.3), r_1_ is the radius of the support circle (mm), r_2_ is the radius of the loaded area (mm) and r_3_ is the radius of the specimen (mm).

For the groups treated with glaze, the stress-moment relation was estimated according to Hsueh’s rigorous solutions.^23^

$$\sigma_{l}=\frac{E_{i}\left(z-z^{*}\right)M}{\left(1-\vartheta_{i}\right)(1+\vartheta )D^{*}}\;(i=1\text{ to }n)$$

i denotes the layer number, z^*^ is the position of the neutral plane, M is the bending moment per unit length, D* is the flexural rigidity and υ is the Poisson’s ratio of the multi-layer.

The stress distribution was estimated with Matlab (MathWorks, Cambridge, UK) to plot the graphic representations of the stresses across the layers, which were based on the estimates of top, bottom and interfacial stresses obtained from Hsueh’s solutions.^23^

### Fractography analysis

Fractured specimens derived from BFS test were evaluated first by optical microscopy (30 ×, Mitutoyo Sul Americana, Suzano, São Paulo, Brazil) and then by Scanning Electron Microscopy (SEM) (Inspect S50 – FEI Worldwide Corporate Headquarters, Hillsboro, OR, USA) to determine the fracture origins.

### Statistical analysis

We used the OpenEpi website (www.openepi.com) to determine the power of the study for the BFS data (95% confidence interval). The distribution of the data was evaluated using the Shapiro-Wilk’s test and homogeneity using the Levene’s test. Two-Way analysis of variance (ANOVA) and Tukey’s test (5%) were performed to compare the effect of glazing region and mechanical aging on BFS between all groups. Statistical analysis was performed using program STATISTIX (Analytical Software Inc., version 8.0, 2003). We considered a 5% significance level.

Weibull analysis considers Weibull modulus (m) and characteristic strength (σ_o_) to evaluate the reliability of the BFS of the material. The characteristic strength indicates the resistance at a failure probability of approximately 63.3%. Weibull modulus indicates the structural homogeneity of the material considering strength distribution. The calculation Weibull modulus and characteristic strength with a confidence interval of 95% were estimated by lnσ_c_–ln [ln 1/(1-F(σ_c_)] diagram (according to ENV 843-5):

$$\ln\ln\left(\frac{1}{1-F(\sigma c)}\right)=\min_{\sigma }c-\mathrm{mln}\sigma$$

Statistical analysis was performed at Minitab software (version 17, 2013, Minitab, State College, PA). The level of significance was 5%.

## Results

### Biaxial flexure strength

The power of 100% was obtained. Statistical assumptions analysis of data indicated a normal distribution of the data (p>0.05). The results of Levene’s test indicated the homogeneity of the data as there was no statistically significant difference between the standard deviations (p=0.196). ANOVA revealed that the factors “Glazing side” (p<0.00001), “Mechanical aging” (p=0.0489) and their interaction (p<0.00001) were statistically significant. Considering the “Glazing side” factor individually, control group showed the lower BFS (737.9^B^ MPa) than the other tested groups (GTC: 1059.0^A^ MPa; GC: 1058.3^A^ MPa; GT: 1025.6^A^ MPa). Moreover, mechanical aging significantly decreased the BFS of Y-TZP (non-aged: 1005.5^A^ MPa; aged: 941.6^B^ MPa).

When we compared all experimental groups, GC (1157.9^A^±146.9 MPa) showed higher BFS than GC-A (958.7±١٢٩.١^BCD^MPa), GT-A (895.0±74.0^CDE^MPa), Control-A (780.8±88.7^DE^ MPa) and control (722.0±145.6^E^MPa) groups. For non-aged groups, there was no significant difference for glazing sides and glazing increased BFS when compared with the control (no glazing). For aged groups, GTC-A (1131.9±128.9^AB^MPa) presented higher BFS than all groups, except to GC-A. Only GTC-A glazing increased BFS when compared with control-A. Mechanical aging significantly decreased BFS of GT and GC groups, whereas BFS of GTC and control groups were not affected (Table 1).

**Table 1:** Mean flexural strength (MPa) with standard deviation, characteristic strength (σo), Weibull modulus (m) and respective CI (95%) for biaxial flexure strength of experimental groups

Aging condition	Glazing side	Group Name	Flexural Strength (MPa)	Weibull Characteristic strength (σ_**o**_) (MPa)	95% CI for σ_**o**_(MPa)	Weibull Modulus (m)	95% CI for m
Non-aged	no glazing	Control	722.0 ± 145.6^E^	779.5^e^	691.6-878.5	5.5^α^	3.2-9.3
	tensile side	GT	1156.1 ± 195.3^AB^	1233.9^ab^	1118.8-1360.8	6.7^α^	4.1-10.9
	compression side	GC	1157.9 ± 146.9^A^	1207.6^a^	1131.7-1288.5	10.7^α^	7.9-14.5
	tensile and compression sides	GTC	986.0 ± 187.4^ABC^	1065.5^abc^	942.6-1204.4	5.4^α^	2.4-12.0
Aged	no glazing	Control-A	780.8 ± 88.7^DE^	820.1^de^	764.8-879.3	9.4^α^	4.5-19.7
	tensile side	GT-A	895.0 ± 74.0^CDE^	928.0^cde^	883.5-974.7	13.3^α^	7.2-24.6
	compression side	GC-A	958.7 ± 129.1^BCD^	1011.9^bc^	936.2-1093.7	8.4^α^	4.6-15.3
	tensile and compression sides	GTC-A	1131.9 ± 128.9^AB^	1185.1^ab^	1110.1-1265.1	10.1^α^	6.8-15.1

Different uppercase letters indicate statistically significant difference for the flexure strength. Different lowercase letters indicate statistically significant difference for the Weibull Characteristic strength. Different greek alphabet letters indicate statistically significant difference for Weibull Modulus. A: mechanical aging.

**Figure 1:**
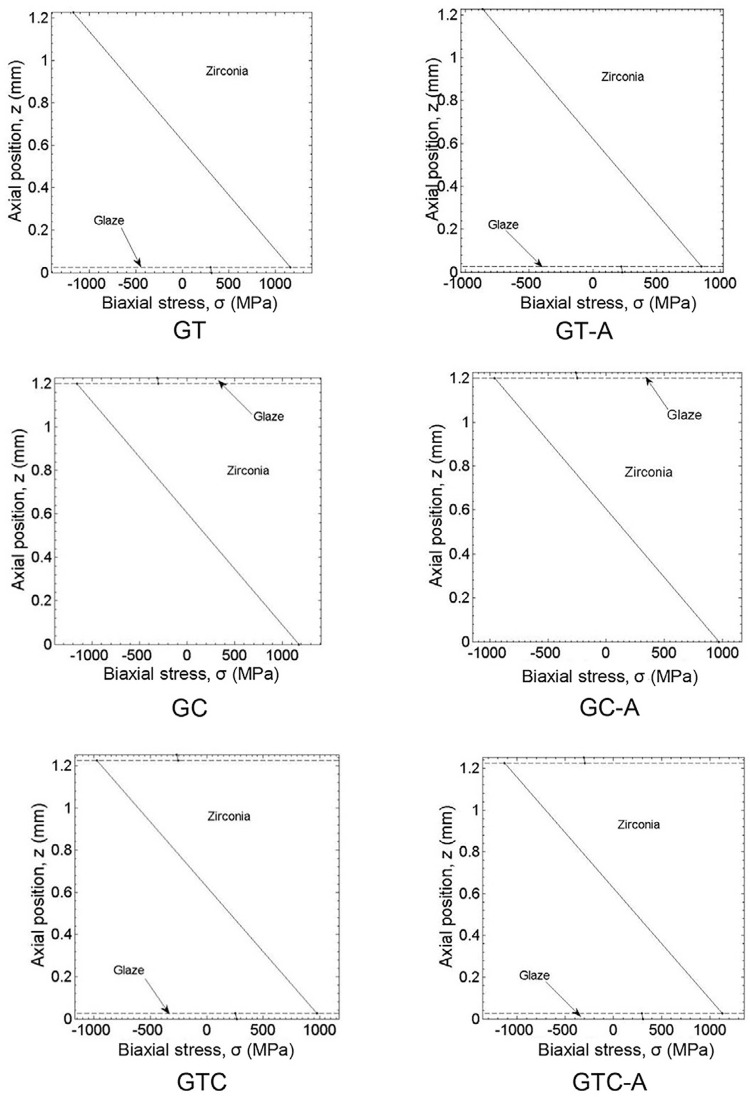
Stresses across the glaze and zirconia layers for the glazed groups. After mechanical ageing, a stress shift to lower strengths is seen on the zirconia surface of groups glazed on one side only

Figure 1 represents the analytical solutions proposed by Hsueh for the piston-on- three ball test of bi- and trilayer specimens. As the maximum tensile stresses required to fracture the specimens always appeared at the lower surface of the zirconia, these values were used for statistical estimates. It is interesting to note that, in the case of glazed zirconia, the stresses across the glaze and zirconia layers can be discriminated, and the maximum tensile stress occurred in the zirconia material at the zirconia/glaze interface, that is, at the bottom surface of zirconia, where cracking imitation occurred.

### Weibull Analysis

**Figure 2:**
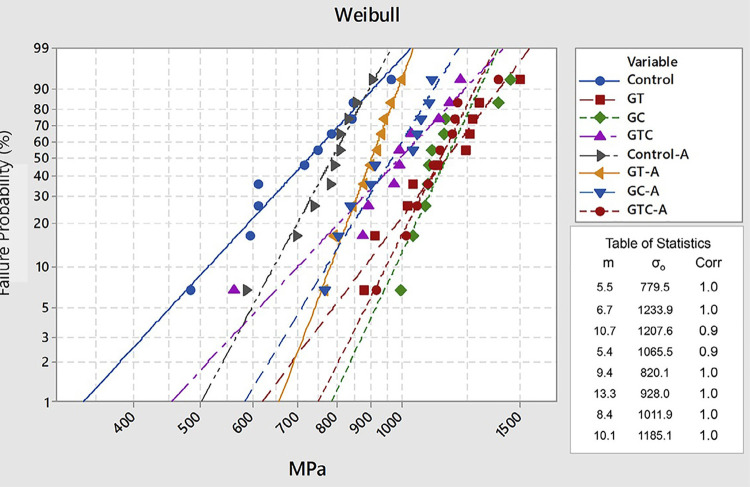
Weibull plot for biaxial flexure strength of Y-TZP

Table 1 and Figure 2 show the results of Weibull analysis are presented. The chi-square test showed no significant difference in the Weibull modulus (m) (p=0.212) of all groups. For characteristic strength (σ_o_), there was significant difference (p<0.0001) between the groups. Group GC (1207.6^a^ MPa) showed significantly higher characteristic strength than control (779.5^e^ MPa), control-A (820.1^de^ MPa), GT-A (928.0^cde^ MPa), and GC-A (1011.9^bc^ MPa). Control presented lower characteristic strength than all groups, except Control-A and GT-A.

### Failure analysis

**Figure 3:**
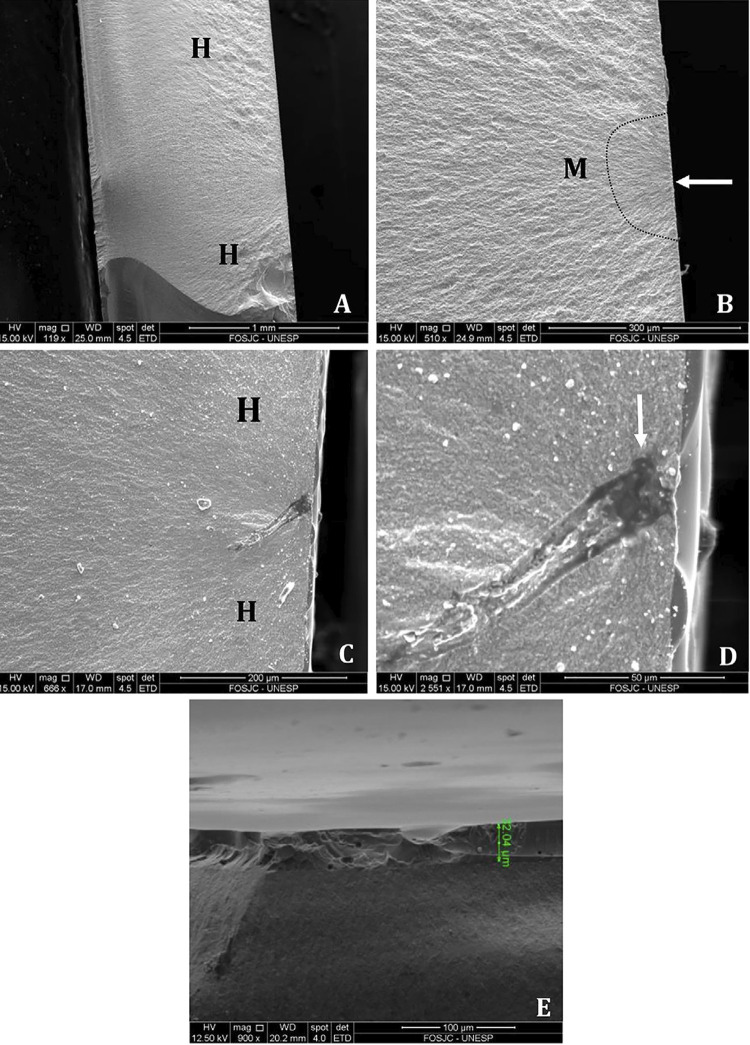
SEM images of fractured disc-shaped ceramic specimens following biaxial flexure strength test. All surfaces seem to present a fracture origin irradiating from the bottom (tensile) zirconia surface (the arrows indicate the failure origin site). The fracture origins of glazed on tensile side (GT) group (119×) (A). H and M are fracture marks that stand for Hackles and Mirror, delimited by the curved line (510×) (B). Failure originated from the surface, close to an internal defect of glaze on tensile and compression side (GTC) group (666×) (C). Close image of internal defect of GTC group (2,551×) magnification (D). Glazed compression side of group GC, where pores are clearly seen, but do not seem to initiate failure (900×) (E)

Figure 3 shows the images of the fractured surfaces. The fractured surfaces showed that the glaze and zirconia layers were bonded without discontinuities. Porosity was observed in the glaze layer. However, fracture origins were observed at the lowest zirconia surface, where surface flaws were typically present.

## Discussion

In an attempt to increase zirconia strength with a procedure that can be easily achieved in a prosthesis laboratory, the zirconia surface was glazed on the tensile and/or compression side to simulate the application of glaze on the inner and occlusal surface, respectively. The first hypothesis, which states that the glaze on tensile and/or compression increase BFS of Y-TZP was partially accepted, since only glaze applied on both side showed higher BFS than control groups for non-aged and aged conditions. Glaze on compression side and tensile side separately increased the BFS of Y-TZP only in non-aged condition. In fact, the glazing of the porcelain tends to create superficial compressive stresses,^24^ which may be similar to those in zirconia-glazed specimens, justifying the high strength values across the glaze layers. This is particularly important because there was no chipping or delamination of the glaze, which is commonly seen in veneered specimens as a result of thermal stresses caused by coefficient of thermal expansion mismatch and zirconia’s poor thermal conductivity.^25^

Higher BFS of most glazed groups is probably due to more favorable distribution of stresses^23^ throughout the tough zirconia layer, as described for graded zirconia.^16^In this case, the reduced modulus in the near-surface regions caused the transfer of the majority of stresses to the inner core material, which is stiffer,^26^ with hardly any in the glaze layer, despite the pores found therein. In our study, the analysis of the stresses across the glaze and zirconia layers for the glazed groups showed that the maximum tensile stress was presented at the bottom surface of the zirconia (tensile side), considering the zirconia/glaze interface. In general, the glaze layer survived cyclic fatigue loading, and fracture occurred in the bottom surface of the zirconia layer, as demonstrated by the fracture origins, probably because it is much stiffer than the glaze layer.^23^

Few information about the effect of glazing side were found in the literature.^27^ Hjerppe, et al.^27^(2010) reported that the application of glaze on tensile side decreased the BFS, whereas glaze on compression side was similar to control (no glazing), in non-aged condition. These results differ from our study, since none of the glazed groups showed BFS lower than control. The authors discussed that the residual stress caused by the cooling rate of the glaze that filled the superficial flaws of the zirconia may increase the probability of fracture. Most of the studies that investigated the effect of glazing on mechanical properties of zirconia applied that glaze in only one side.^14,28,29^ The authors reported increased,^14^ decreased^30^ or similar^28,29^ mechanical behavior when compared with non-glazed zirconia specimens. Further studies are necessary to evaluate the application of glaze on tensile and compression side of Y-TZP, considering the promising results of our study for mechanical properties and the advantages for bond strength to resin cement.^19^

The second hypothesis that mechanical aging decreases BFS for all groups was partially accepted, since aging decreased BFS of GT and GC groups, whereas control and GTC groups showed similar BFS to non-aged and aged conditions. The decrease in strength of specimens glazed only on the tensile or compression sides after mechanical aging in water is noteworthy. Stress distribution showed lower stress values in these groups after mechanical aging. Therefore, asymmetric stress distributions caused by glazing only in one side and the presence of water may be responsible for such decrease in strength, which did not occur in the groups glazed on both sides. However, this needs further investigation.

Previous studies have reported that mechanical aging did not significantly decrease the mechanical properties of 3Y-TZP.^31-34^ The decrease of the flexural strength was reported with more aggressive protocols of mechanical cycling;^35^ however, all studies used a monolithic zirconia, without glaze application. The parameters used in the current study are consistent with that of Wiskott, Nicholls and Belser^36^ (1995), which reported that a minimum of 10^6^ cycles should be used to simulate the masticatory function. Moreover, the mean natural chewing forces range from 70.6–146.1 N.^37^ Thus, the 84 N load performed in our study is within that range. In our study, mechanical aging was performed with samples immersed in water, which can negatively influence the mechanical properties of the samples.^38^ Considering that mechanical cycling was performed to simulate the aging caused by chewing, but without necessarily causing fracture of the samples, the parameters of 1.2×10^6^ mechanical cycles, 3 Hz, 84 N, in water were adopted. Further studies with a higher number of mechanical cycles and loads are important to investigating the mechanical performance of zirconia, simulating more adverse clinical conditions such as the presence of parafunctional habits.

We performed the Weibull analysis to compare the reliability of the material, depending on the treatments. The Weibull moduli were not significantly different (shown by the overlapping of the confidence intervals), which probably means that glazing could not change the original defects, such as by healing of surface defects. Moreover, in aged and non-aged conditions, glaze on both sides increased characteristic strength. Stress distribution possibly worked differently in glaze groups and shifted the materials’ strength to higher values.^16^ The current study performed this analysis with a sample size smaller than the conventional recommendation of 30 specimens per group.^39^ Several studies have also performed this analysis with a sample size of n=10.^40-44^ Quinn and Quinn^39^(2010) stated that bends or wriggles in the trend line could be a consequence of small sample sizes; however, this was not seen herein.

Increased load-bearing capacity^45^was observed in full-contour single crowns after However, we emphasize that glaze layers can be worn off or partially removed with hydrofluoric acid etching.^46^ Thus, further studies should be done with full crowns varying the sides of glaze application. Considering the limitations of our study, glazing the occlusal and cementation surfaces of zirconia may improve the long term performance of monolithic zirconia.

## Conclusion

Based on our research, we conclude that:

- The application of glaze on both tensile and compression sides improved the flexural strength of 3Y-TZP, regardless of the mechanical aging;
- The origin of fractures was on the tensile surface of zirconia and not on the glaze layer.

### Clinical relevance

Glazing of tensile and compression areas seems to be a promising approach to improve zirconia ceramic mechanical properties and long-term.
